# Behavioural determinants of malaria risk, prevention, and care-seeking behaviours among forest-goers in Cambodia

**DOI:** 10.1186/s12936-022-04390-5

**Published:** 2022-12-02

**Authors:** Sochea Phok, Kemi Tesfazghi, Andy Tompsett, Boukheng Thavrine, Po Ly, Saad El-Din Hassan, Avery Avrakotos, Jim Malster, Erica Felker-Kantor

**Affiliations:** 1 Population Services International, Phnom Penh, Cambodia; 2 Population Services International, Washington, DC USA; 3 National Centre for Parasitology, Entomology and Malaria Control, Phnom Penh, Cambodia; 4 US President’s Malaria Initiative, United States Agency for International Development, Phnom Penh, Cambodia; 5 US President’s Malaria Initiative, United States Agency for International Development, Washington, DC USA

**Keywords:** Forest-goers, Malaria, Long-lasting insecticide-treated nets, Care-seeking, Cambodia

## Abstract

**Background:**

Cambodia has made significant progress towards achieving malaria elimination by 2025. Cases continue to decrease and are primarily concentrated in forested areas. Forest-goers are most at risk of malaria due to their proximity to the forest, poor sleeping conditions, frequent mobility, and distance from health services. Consistent use of long-lasting insecticidal nets or hammock nets (LLINs/LLIHNs), early diagnosis and treatment of cases are central to reducing disease burden. The aim of this study was to understand forest-goers’ knowledge, attitudes, and practices related to malaria prevention and care-seeking, and to identify key behavioural determinants of LLIN/LLIHN use and prompt care-seeking within 24 h of developing a fever.

**Methods:**

A mixed-methods study design consisting of a cross-sectional survey and qualitative in-depth interviews was implemented in two Cambodian provinces. Survey participants (N = 654) were recruited using respondent driven sampling. Interview participants (N = 28) were selected using purposive sampling. Findings from the survey were analysed using univariate and bivariate analysis and multivariate weighted logistic regression. Interviews were coded and analysed using thematic content analysis.

**Results:**

All study participants had heard of malaria and 98% knew that malaria was transmitted by mosquitoes. LLIN/LLIHN ownership was high (94%). Although 99% of participants perceived LLIN/LLIHN use as an important malaria prevention measure, only 76% reported using one during their last visit to the forest. Only 39% of survey participants who reported seeking care did so within the recommended 24 h from fever onset during their last febrile illness. Among all study participants, 43% did not seek any healthcare during their last febrile episode. In controlled regression models, perceived community social norms were significantly associated with LLIN/LLIHN use (OR: 2.7, 96% CI 1.99–2.64) and care-seeking within 24 h of fever onset (OR: 1.7, 95% CI 1.00–2.88). Social support from other forest-goers was also significantly associated with LLIN/LLIHN use (OR: 4.9, 95% CI 1.32–18.12).

**Conclusions:**

Study findings are consistent with other studies on LLIN/LLIHN use and care-seeking behaviours. While rates of LLIN/LLIHN ownership were high among the study population, rates of use were not as high. More concerning were the delayed care-seeking behaviours. Social behaviour change activities should incorporate social norms and social support as mechanisms for behaviour change given the identified positive correlations with LLIN/LLIHN use and prompt care-seeking.

## Background

Over the past decade, Cambodia has made significant progress towards elimination of malaria. The national malaria burden decreased from 100,000 cases in 2010 to less than 10,000 in 2020 [1]. In 2021, there were 4329 confirmed cases. Deaths from malaria have also decreased, declining from an estimated 151 reported deaths in 2010 to zero since 2018 [1]. While malaria is still endemic across the country, transmission is predominantly localized in forested areas [2]. Cases of *Plasmodium falciparum* have declined considerably in the country, dropping from 61% of all malaria cases in 2015 to just 16% in 2019. In 2021, *Plasmodium vivax* represented 91.5% of all cases [3, 4].

In Cambodia, mobile and migrant populations (MMPs) are most at risk for malaria as they frequently move between low and high transmission areas and have limited access to health services and information [5, 6]. Forest-goers have been identified as the MMP group with the highest exposure to malaria due to their proximity to forests, poor sleeping conditions (e.g., hammocks, tents), frequent mobility, and distance from health services. They are also more at risk than village or urban populations as the mosquitoes that carry malaria are more prevalent in forested areas with day and evening biting times. In 2013, malaria prevalence was 5.4% among forest-goers compared to 1.2% among the general population [3].

To accelerate progress towards malaria elimination, the National Center for Parasitology, Entomology and Malaria Control (CNM) launched the Malaria Elimination Action Framework (MEAF) 2016–2020 [3]. The MEAF proposed a wide range of elimination activities with a specific emphasis on targeting MMPs. The proposed intervention strategies included universal coverage of case management services to ensure diagnosis of all suspected cases and effective treatment of all confirmed cases, scale-up of vector control measures, such as widespread distribution of long-lasting insecticidal nets and hammock nets (LLIN/LLIHN), improved surveillance, and implementation of comprehensive social behaviour change (SBC) activities to increase LLIN/LLIHN use and care-seeking within 24 h of developing a febrile illness.

Social behaviour change communication is an effective tool to influence knowledge about malaria prevention and healthcare-seeking practices [7, 8]. For example, a study in Cambodia reported that intense behaviour change communication (BCC) was effective at increasing knowledge about local malaria risk factors and malaria drug resistance [9]. The same study also found a significant association between BCC exposure and the frequency of discussion about malaria in the family [9]. Another study in Zambia found that exposure to BCC on mosquito net use accounted for a 29% increase in insecticide-treated net (ITN) use among households that already owned an ITN [10]. A pilot study conducted in Zimbabwe implemented a participatory quality improvement approach with the aim of improving malaria case management and showed that malaria case investigation rates improved from 55% at baseline to 85% post intervention in groups that were exposed to the SBC intervention [11].

Formative research is a critical first step to designing effective SBC activities [12]. The goal of formative research is to develop a better understanding of the target population, specifically their knowledge, attitudes, and practices (KAP) in relation to the topic of study, and their motivations and barriers to adopting and changing behaviour [13]. In 2020, in support of the CNM’s National Strategic Plan for Elimination of Malaria in the Kingdom of Cambodia, Population Services International (PSI), as part of its Promoting Healthy Behaviours Activity (PHB) funded by USAID and the United States President’s Malaria Initiative (PMI), conducted a formative research to assess forest-goers’ KAP in relation to malaria prevention and care-seeking behaviours and to identify key behavioural determinants correlated with LLIN/LLIHN use and care-seeking within 24 h of developing a febrile illness. The findings from the formative research will inform the development—or tailoring of—SBC activities to promote malaria prevention and care-seeking behaviours among forest-goers.

This study presents the key findings from the formative research, summarizing malaria KAP of forest-goers, and highlights the main behavioural determinants that were correlated with LLIN/LLIHN use and care-seeking within 24 h of fever onset.

## Methods

### Study design

This study used a mixed-methods design, employing qualitative in-depth interviews (IDIs) and a cross-sectional quantitative survey from August to September 2020, which is rainy season and a period of high transmission. Cambodia’s National Center for Human Research Committee and PSI Research Ethics Board provided ethical approval. Written informed consent was obtained from all study participants. While RDS is similar to snowball sampling in that participants recruit or refer other participants to the study, RDS is based on the principals of Markov chain theory and network theory. RDS relies on multiple waves of peer-to-peer recruitment and applies statistical adjustment to approximate random sampling and produce unbiased population estimates.

### Study area

**Fig. 1:**
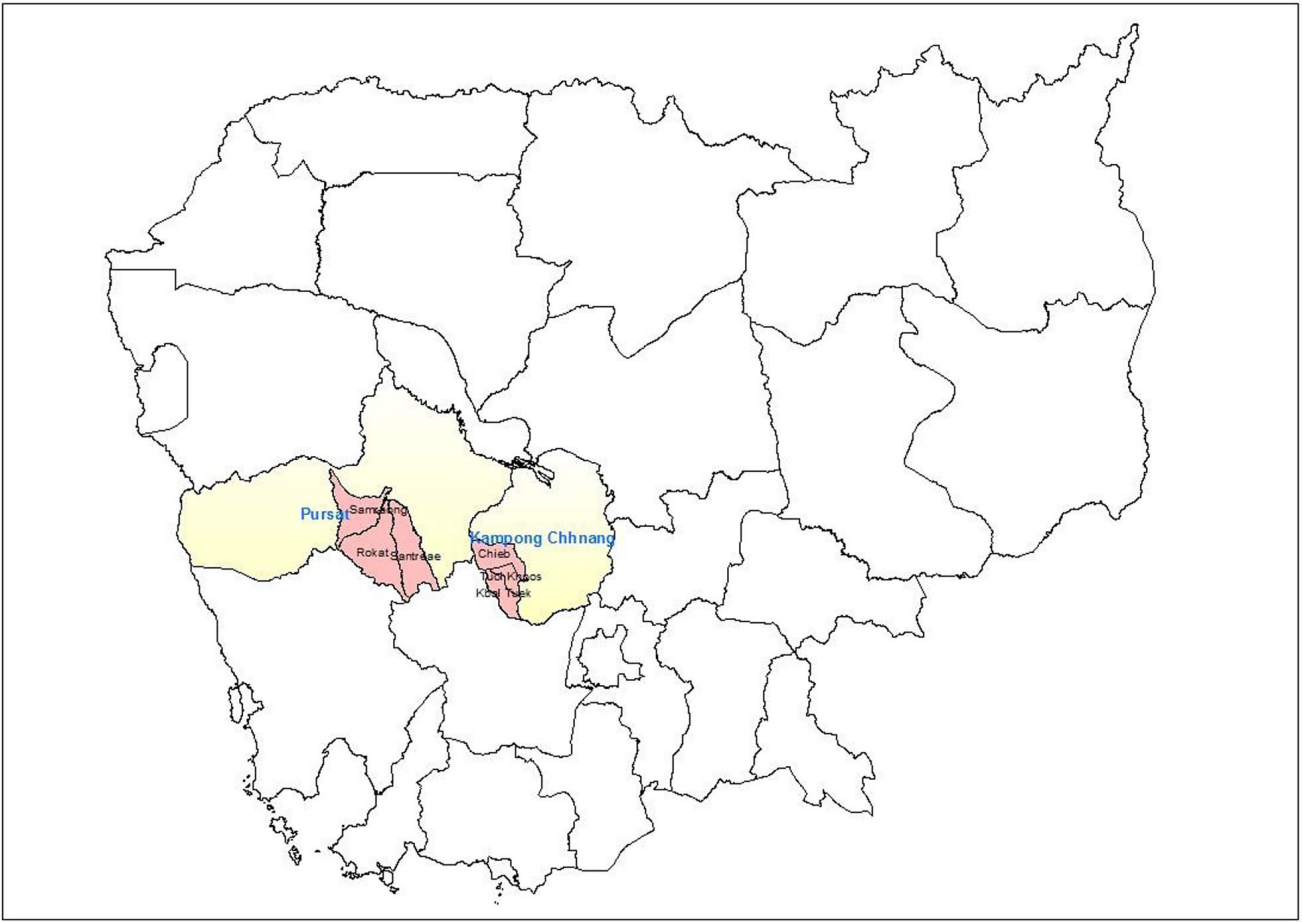
Map of study provinces and communes

Study locations were selected using a multi-staged sampling approach. Two operational provinces were first selected, Kampong Chhnang and Pursat, based on recommendations from CNM and the U.S. PMI (Fig. 1). Within each province, one operational district was then selected, Teuk Phos in Kampong Chhnang province and Phnom Kravanh in Pursat province were selected due to high incidence of malaria, proximity to the forest, and a large population of forest-goers. Next, three communes in each district were selected using probability proportional to size sampling. Within the six communes, 27 villages were grouped into 16 clusters for sampling.

### Sample size

Sample size was calculated using a proportion of 50%, a confidence interval of 95%, a margin of error of 5%, and a design effect of 2, resulting in a target sample size of 675.

### Study population and sampling approach

In Cambodia, forest-goers are considered a hard-to-reach population due to their mobile and migratory status. Due to this and the lack of a sampling frame, respondent-driven sampling (RDS) was used to recruit study participants from the 16 clusters for the quantitative survey. RDS is a chain referral sampling method which relies on social network dynamics to sample hard-to-reach and hidden populations and is an established method for obtaining samples when a sampling frame is not available [14–17].

Men and women forest-goers were eligible to participate in the study if they met the following criteria: (1) had spent at least one night in the forest in the 30 days prior to the study; (2) were 18 years or older; (3) had a febrile illness event during the past 3 months; and (4) provided written informed consent.

In RDS, sampling begins by purposively selecting seeds (initial recruits) from the study population to recruit peers from their social networks. Sixteen initial seeds were non-randomly selected based on pre-established selection criteria through collaboration with key informants including village chiefs, village malaria workers (VMWs), and community leaders. Each seed was administered the survey and then provided with three recruitment coupons to recruit the first wave of participants who met eligibility criteria. Subsequent waves of participants who completed the survey also received three recruitment coupons to recruit additional participants. Participants were instructed to provide the coupons to members of their social network who met the study eligibility criteria. Participants received a monetary incentive (10,000 Cambodian Riel equivalent to $2.5 US dollars) for each eligible person they recruited who consented to participate in the survey and an incentive of the same amount for completing the survey. To ensure confidentiality, participant coupons and surveys were identified using a unique study identification number. A coupon manager system was used to monitor recruitment and incentive distribution. Recruitment continued until the estimated sample size was reached.

Participants for the qualitative IDIs were selected from the study areas using purposively sampling. IDI participants were from the same study area where the quantitative survey was administered and had participated in the quantitative survey.

### Data collection

#### Quantitative survey

The quantitative survey included sections on socio-demographics, the ID Poor Programme (a programme established by Ministry of Planning to support the poor through social assistance interventions in health and other sectors) forest-going frequency and purpose, knowledge of malaria transmission, malaria prevention behaviours, LLIN/LLIHN ownership and use, knowledge and use of health care services, preferences for health service providers, and information regarding the most recent fever episode [18]. In addition, the survey measured specific behavioural determinants that were identified a priori as potential barriers and facilitators of malaria prevention behaviours. The behavioural determinants were selected based on the Opportunity, Ability, and Motivation Behaviour Change Framework which classifies determinants as internal, social or structural (Table 1) [19]. The survey was administered in Khmer and took 45–60 min to complete.

**Table 1:** Opportunity, ability, and motivation behaviour change framework

Behavioural determinant	Level	Definition
Opportunity		
Availability	Structural	The extent to which the promoted product or service is found in a predefined area
Social norms	Social	The behavioural standards which exist in the community for an individual to follow
Quality of care	Structural	The extent to which the promoted service is of high value
Ability		
Knowledge	Internal	True facts accumulated through learning about objects, actions and events
Social support	Social	The assistance that an individual gives/receives
Self-efficacy	Internal	The belief that an individual is able to perform a promoted behaviour effectively or successfully
Motivation		
Threat	Internal	The perception of the severity of the problem (including physical, psychological, or economic harm)
Belief	Internal	A perception about an object or event, which may or may not be true
Outcome expectation	Internal	Perception of whether a promoted product, service, or behaviour is effective in fulfilling its purpose as intended

#### In-depth interviews

Twenty-eight IDIs were conducted with forest-goers who met the study eligibility criteria. Purposive sampling based on LLIN/LLIHN use during the last visit to the forest was used to select IDI participants. All IDI participants were from the same study area where the quantitative survey was administered and had participated in the quantitative survey. Half the sample (n = 14) had slept under an LLIN/LLIHN during their last visit to the forest and half (n = 14) had not. Interviewees were recruited by community leaders and key informants. Interviews were conducted in Khmer by two trained interviewers at forest-goer shelters or camps. Interviews were audio-recorded and lasted an average of 60 min. Interview questions focused on forest-goer characteristics, forest-going activities, malaria knowledge and prevention practices, experiences with febrile illness, experiences seeking health care, perceptions of health care quality, and behavioural determinants that may influence care-seeking. Interview participants received a non-monetary incentive for their participation.

### Data analysis

#### Quantitative survey

Quantitative data were analysed using STATA version 12. Using the RDS survey command, sampling weights were created for each variable by calculating each participant’s probability of selection proportional to their social network size. Weights were applied to each variable during statistical analysis. Descriptive statistics were used to summarize socio-demographic characteristics, malaria KAP, and prevention and care-seeking behaviours. Bivariate analysis (chi-square) was performed to examine associations between behavioural determinants thought to influence LLIN/LLIHN use and care-seeking within 24 h of developing a febrile illness. Behavioural determinants that were significantly associated with LLIN/LLIHN use or care-seeking within 24 h of developing a febrile illness, were further analysed in multivariate logistic regression models.

Multivariate logistic regression was performed to estimate crude and adjusted odds ratios and 95% confidence intervals. Variables that created 10% or greater difference between the unadjusted and adjusted effects and that were not considered mediators were considered confounders and controlled for in the final model. Additionally, covariates with significant statistical associations to both the outcome and exposure in bivariate analyses were included in the regression models, in addition to variables that were not statistically significant but that are important theoretical confounders. Multicollinearity was assessed by examining correlations between covariates and variance inflation factors (VIFs). The model did not include any measure that had a value of 7.0 or greater for VIF. Model fit was assessed using goodness of fit statistics. Standard errors, 95% confidence interval, and unless otherwise stated, a p-value of < 0.05 was used to define statistically significant associations [20].

#### In-depth interviews

A content analysis approach was used to analyse the IDIs [21]. The audio-recorded interviews were first transcribed verbatim. Then, using an initial codebook which was developed with a priori codes based on the study objectives, two trained researchers conducted line-by-line coding of the interview transcripts. Themes were identified throughout the iterative coding process using an applied thematic approach and relevant data were extracted and categorized based on the thematic content. The themes and coded data were explored for patterns and interpreted based on study context and used to further inform findings from the quantitative survey.

## Results

Quantitative and qualitative findings are integrated and presented together by theme.

### Sociodemographic characteristics

Table 2 presents the weighted sociodemographic characteristics of the study population. A total of 654 participants were included in the study. Most forest-goers were 30 years or older (67%), male (85%), and married (86%). More than half (55%) completed primary school, 23% completed secondary school, and 22% had no schooling or attended some school but did not complete any level. Approximately 25% of forest-goers participated in the Identification of Poor Households Programme (ID Poor). Forty-five percent of participants reported farming as their primary occupation, followed by wood cutting (40%), worker/labourer in forest activities (9%), and other forest-related work including collecting resins, mushrooms, and bamboo sprouts (6%). During their last visit to the forest, participants spent an average of nine nights [range 1–30] in the forest.

**Table 2:** Sociodemographic characteristics of study sample

	Unweighted sample (n)	Weighted population proportion	Bootstrapped 95% CI
Age (years)			
18–19	21	3.8	(2.4–5.7)
20–24	67	10.4	(8.2–13.0)
25–29	119	18.4	(15.6–21.6)
30–34	123	20.4	(17.4–23.7)
35–39	129	19.4	(16.6–22.6)
40–44	71	10.1	(8.1–12.6)
45–49	51	7.7	(5.9–9.9)
50+	73	9.9	(7.9–12.3)
Sex			
Male	566	85.0	(82.0–87.7)
Female^a^	88	15.0	(12.3–17.9)
Marital status			
Married	560	85.9	(83.0–88.4)
Single	83	12.5	(10.2–15.3)
Divorced	11	1.5	(0.8–2.7)
ID poor card^b^			
Yes	162	26.5	(23.2–30.1)
No	492	73.5	(69.8–76.7)
Education^c^			
Never attended school	87	14.8	(12.2–17.9)
Attended but did not complete any level	47	7.1	(5.3–9.3)
Completed primary	361	55.1	(51.2–58.9)
Lower or secondary	157	22.9	(19.9–26.2)
Higher	1	0.0	(0.00–0.3)
Primary occupation^c^			
Farming	295	45.2	(41.4–49.1))
Worker/labour seller in forest activities	260	8.6	(6.7–11.1)
Wood cutting	57	39.9	(36.3–43.7)
Forest-related work	40	6.0	(4.4–8.1)
Other	0	0.0	(0.004–0.7)
Length of stay during last visit to the forest			
Short (≤ 7 nights)	398	65.1	(61.4–68.5)
Medium (8–15 nights)	110	13.3	(11.1–16.0)
Long (> 15 nights)	146	21.6	(18.6–25.0)

% of any variable based on < 10% missing

CI confidence interval

^a^Unweighted data due to not large enough sample as required by RDS analysis

^b^ID Poor is a government programme that supports the poor through social assistance interventions in health and other sectors

^c^Frequencies may not add up to 100% due to missing/non-response

### Malaria knowledge and perception of risk

All study participants had heard of malaria and 98% knew that malaria was transmitted by mosquitoes. Fourteen percent of forest-goers thought that one could become infected with malaria by drinking stream water. This finding was also identified in the IDIs. According to one IDI participant:

*“Malaria is transmitted from mosquitoes, the female Anopheles. However, as far as I know, for forest-goers like us, people get malaria from drinking unclean water when they are in the forest. I mean sometimes the water that we drink contains rusty substances because we can see the red color.” [Forest-goer, 50 years, Ksat Borey Village]*

Malaria was perceived as a common disease and study participants believed that most people, including their family, friends and community members had been infected with malaria at one time. Most forest-goers, 79%, perceived malaria as harmful and 40% believed it was hard to cure. While forest-goers perceived that they were at the greatest risk for malaria, they also recognized that other villagers could become infected, and that malaria was dangerous not only to individual health but also to family livelihood. As one individual explained, contracting malaria could prevent forest-goers from doing their daily job and in turn, impact their earnings and family income:

*“Yes, everyone is concerned about getting malaria since it’s not only forest-goers but also villagers who can get malaria. When we have malaria, we cannot get 100% cured and we keep on relapsing, and this results in lost time and income for the family.” [Forest-goer, 27 years, Prek Village]*

### Prevention behaviours and practices

Table 3 presents weighted descriptive statistics of malaria prevention preferences, behaviours, and practices. The three preferred methods for preventing malaria were LLINs/LLIHNs (70%), mosquito repellent (55% spray, 46% lotion), and mosquito coils (46%). Although mosquito repellent was reported as a preferred method for preventing malaria, forest-goers’ knowledge of repellents, including where to obtain repellents, was limited. Repellent use was also low with only 9% of participants reporting that they used repellent regularly as a prevention measure.

**Table 3:** Malaria prevention behaviours and practices among forest-goers in Cambodia

	Unweighted sample (n)	Weighted population proportion	Bootstrapped 95% CI
Preferred methods for malaria prevention^a^			
LLIN/LLIHN	471	70.2	(66.5–73.6)
Spray repellent	365	54.8	(50.9–58.6)
Lotion repellent	308	45.9	(42.1–49.7)
Mosquito coil	303	45.7	(42.9–49.5)
Soaked dress/cloth	217	34.8	(31.3–38.6)
Patch repellent	119	20.6	(17.5–24.1)
Prevention medication	98	17.3	(14.5–20.6)
Mosquito killer spray	23	2.4	(2.3–5.2)
Repellent use			
Used in the past 3 months	59	9.1	(7.2–11.6)
Used, but more than 3 months ago	46	7.6	(5.8–10.1)
Never use	549	83.2	(80.0–85.9)
LLIN/LLIHN availability			
Household owns at least one LLIN/LLIHN	622	94.0	(91.7–95.7)
LLIN/LLIHN can be brought to forest	583	94.7	(92.8–96.1)
Obtained a new LLIN/LLIHN in last year	561	88.3	(85.3–90.8)
LLIN/LLIHN use during the last visit to the forest			
Did not use a net	112	16.1	(13.6–19.1)
Slept under LLIN/LLIHN some or every night	483	75.4	(72.0–78.5)
Slept under untreated net	59	8.5	(6.6–10.8)
Reasons for not using LLIN/LLIHN regularly			
No mosquitos in the area	12	8.3	(4.7–14.3)
Did not bring LLIN/LLIHN to forest	25	19.3	(13.3–27.4)
Do not have LLIN/LLIHN	24	20.7	(14.2–29.2)
Too hot to sleep under	52	50.6	(41.3–59.9)
LLIN/LLIHN is too old	11	11.6	(6.5–19.9)
Bad smell	4	4.1	(1.5–10.5)
Other	14	7.9	(4.6–13.2)

% of any variable based on < 10% missing

LLIN/LLIHN long-lasting insecticidal net/long-lasting insecticidal hammock net, CI confidence interval

^a^Multiple response question

Findings from the IDIs also revealed that fire was a popular method for preventing mosquito bites. Participants explained that forest-goers made fires even when using an LLIN/LLIHN at night because they believed that fires repelled mosquitoes:

*“In addition to the LLIHN, we build a fire, and with the smoke from the fire and a blanket to cover our body this is good enough to sleep through the night. I usually use both LLIHN and make a fire.” [Forest-goer, 29 years, Khsat Borey Village]*

### LLIN/LLIHN availability

Nearly 95% of forest-goer households owned at least 1 LLIN/LLIHN. Among forest-goers whose household owned at least 1 LLIN/LLIHN, 95% were able to bring it to the forest. Slightly more than 85% of study participants had obtained a new LLIN/LLIHN in the past year. The average number of LLINs per household was 2 [range 1–10] while the average number of LLIHNs was 1 [range 1–5] (Table 3).

IDIs revealed that the main reasons forest-goers did not have or obtain an LLIN/LLIHN were due to forest-goers not being in the village during net distribution. Another reason which caused them to not have an LLIN/LLIHN while in the forest was due to the LLIN/LLIHN being seriously damaged or torn by nails, thorns, or sticks during the last time of use.

### LLIN/LLIHN use

Nearly all forest-goers who participated in the quantitative survey (99%) reported that it was necessary to sleep under an LLIN/LLIHN when in the forest and a similarly high proportion reported that treated nets were an effective method for preventing malaria. Despite this, only 75% of study participants reported that they slept under an LLIN/LLIHN every night during their last visit to the forest, 9% slept under an untreated mosquito net, and 16% did not use an LLIN/LIH every night. Forest-goers who did not use a treated net/hammock net every night, provided several reasons for not regularly using an LLIN/LLIHN during their last visit to the forest: discomfort due to heat (51%), not owning an LLIN/LLIHN (21%), not bringing an LLIN/LLIHN to the forest (19%), low perceived mosquito density (8%), perceived poor quality of LLIN/LLIHN (12%), smell (4%), and other (8%) (Table 3).

Findings from the qualitative interviews supported the quantitative findings. Participants who did not use an LLIN/LLIHN at all during their last visit to the forest explained that sleeping under an LLIN/LLIHN was uncomfortable due to heat and smell:

*“Yes, I feel too uncomfortable when I sleep under a net because it makes me feel hot.” [Forest-goer, 25 years, Krasang Dosleung Village]*

*“I can’t stand to the smell from that LLIN/LLIHN, it makes me feel unwell when I smell it and that’s why I decided not to sleep under it.” [Forest-goer, 50 years, Khsat Borey Village]*

Qualitative findings also indicated that seasonality was an important factor influencing LLIN/LLIHN use. They explained that LLIN/LLIHN use typically increased during the rainy season because there were more mosquitoes. During the dry season, however, LLIN/LLIHN use often decreased because the weather was hot and there were fewer mosquitoes. One participant explained:

*“During the rainy season, there are a lot of mosquitoes and people use a net a lot, but in the dry season not many of us use nets for sleeping. If we go somewhere which is not close to water during the dry season, we do not need to use a net because there are fewer mosquitoes. We will use a net only when we camp near a source of water.” Forest-goer, 22 years, Tankhasch Village]*

Another participant described that even if nets were available many forest-goers did not know how to use them properly and developed rashes which deterred them from using nets in the future:

*“Some of the people don’t know how to use it properly. They just pull the net from the bag and use it for sleeping, then they get a rash on their whole body. It is like applying red pepper on your body, but for me it is fine because I wash it first after getting it from the distribution center and before using it.” [Forest-goer, 33 years, Santrae Village]*

### Behavioural determinants of LLIN/LLIHN use

The following behavioural determinants were correlated with LLIN/LLIHN use in bivariate analysis: social norms, social support, and beliefs (Table 4).

**Table 4:** Bivariate analysis of behavioural determinants associated with LLIN/LLIHN use during the last visit to the forest among forest-goers in Cambodia

	LLIN/LLIHN use			No LLIN/LLIHN use			Pearson Chi-square statistic	p-value
	Unweighted sample (n)	Weighted population proportion	Bootstrapped 95% CI	Unweighted sample (n)	Weighted population proportion	Bootstrapped 95% CI		
Social norms*—perceived norms that members of the community sleep under LLINs/LLIHNs to prevent malaria*								
Yes	417	75.4	(72.0–78.5)	109	19.4	(15.0–22.8)	50.0	0.00
No	66	55.6	(45.0–62.0)	62	46.4	(38.1.2–55.1)		
Social support—*received social support from other forest-goers to use LLIN/LLIHN while in forest*								
Yes	432	77.3	(73.7–80.5)	138	22.8	(19.6–26.3)	8.6	0.00
No	51	62.6	(51.9.0–72.2	33	37.4	(27.8–48.1		
Beliefs—*belief that LLINs/LLIHNs will NOT harm your health*								
Yes	193	70.9	(65.4–75.9)	86	29.1	(24.2–34.6)	5.5	0.02
No	290	78.7	(74.4–82.5)	85	21.3	(17.5–25.6)		

LLIN/LLIHN long-lasting insecticidal net/long-lasting insecticidal hammock net, CI confidence interval

#### Social norms, social support, and belief

Perceived community social norms supportive of LLIN/LLIHN use among forest-goer communities were significantly correlated with individual LLIN/LLIHN use while in the forest (Chisq = 50.0, p < 0.00). Similarly, support from other forest-goers for LLIN/LLIHN use was correlated with LLIN/LLIHN use during the last visit to the forest (Chisq = 8.6, p < 0.00). Among forest-goers who received encouragement from other forest-goers to sleep under an LLIN/LLIHN (87%), 77% used an LLIN/LLIHN during their last visit to the forest and 23% did not. Beliefs about the potential negative health impacts caused by the chemical on the LLIN/LLIHN were also correlated with LLIN/LLIHN use (Chisq = 5.5, p < 0.02). Among forest-goers who did not believe LLIN/LLIHN to be harmful, 71% used an LLIN/LLIHN during their last visit to the forest and 29% did not.

Results from the crude and adjusted multivariate logistic regression are presented in Table 5. In both the crude and adjusted models, LLIN/LLIHN use was significantly correlated with perceived supportive community social norms for LLIN/LLIHN use. Forest-goers who perceived that LLIN/LLIHN use was a normative behaviour in the community were 2.7 times (95% CI 1.99–3.64, p-value: 0.00) more likely to have used an LLIN/LLIHN during their last visit to the forest compared to those who did not perceive LLIN/LLIHN use as a normative community behaviour, adjusting for covariates and confounders. Social support was also significantly correlated with LLIN/LLIHN use. In adjusted models, forest-goers who received social support from other forest-goers to sleep under an LLIN/LLIHN while in the forest had a higher odds of LLIN/LLIHN use during their last visit to the forest compared to those who did not receive social support (OR: 1.7, 95% CI 1.00–2.88, p-value: < 0.05).

**Table 5:** Weighted crude and adjusted odds ratio and 95% confidence intervals of LLIN/LLIHN use during the last visit to the forest regressed on perceived community social norms of LLIN/LLIHN use and social support of net use among forest-goers in Cambodia

	Unadjusted OR	95% CI	P-value	Adjusted OR^a^	95% CI	p-value
Perceived community social norms						
Yes	3.25	(2.47–4.27)	0.00	2.69	(1.99–3.64)	0.00
No (ref)						
Social support						
Yes	2.03	(1.25–3.27)	0.00	1.70	(1.00–2.88)	< 0.05
No (ref)						

LLIN/LLIHN long-lasting insecticide treated or long-lasting insecticide treated hammock net, OR odds ratio, CI confidence interval

^a^Controlled for age, sex, marital status, education, ID poor, exposure to malaria social behaviour change campaign within the past 6 months, number of nights spent in the forest during last visit, number of forest-goers in the family, availability of LLIN/LLIHN in household

### Health care-seeking behaviours

During their last febrile illness event, 34% of participants suspected that they had malaria, 28% a cold, 17% a general fever, 10% typhoid, 5% the flu, and 5% another illness. Among all participants, 21% were ‘early care seekers’ (< 24 h), 20% were ‘delayed care seekers’ (between 24–48 h), 16% were ‘wait and see’ see care seekers (> 48 h) and 43% did not seek care at all (categorization not shown in table). Fifty-seven percent of forest-goers sought care outside the home during their last febrile illness. Among the 53% that sought care outside the home, 39% sought care within 24 h of developing a fever, 34% sought care within 48 h, and 27% sought care > 48 h. Among the 43% of forest-goers who did not seek care outside the home during their last febrile illness, 37% used home treatment, 77% thought their illness was not serious, and 29% were in the forest at the time of becoming sick so they could not seek care (multiple response options possible). Table 6 presents weighted descriptive findings on health care-seeking behaviours.

**Table 6:** Perceived illness and health care-seeking behaviours among forest-goers in Cambodia

	Unweighted sample (n)	Weighted population proportion	Bootstrapped 95% CI
Perceived type of illness among forest-goers during last febrile illness			
Malaria	222	33.8	(30.0–37.5)
Dengue	6	0.9	(0.4–2.1)
Typhoid	69	9.7	(7.7–12.1)
Cold	179	27.8	(24.4–31.4)
Flu	38	5.1	(3.7–6.9)
Just fever	108	16.9	(14.2–20.1)
Other	31	5.4	(3.7–7.5)
Sought care outside home			
Yes	393	56.8	(52.9–60.6)
No	261	43.2	(39.4–47.0)
Reasons for not seeking care outside home			
Not serious	185	76.4	(71.2–80.9)
Use home treatment	117	36.8	(31.3–42.6)
In forest when got sick	85	28.4	(23.4–34.0)
Sought health care services outside the home within 24 h of fever onset			
Yes	153	38.7	(30.6–40.1)
No	240	61.3	(59.9–69.4)
First source of care among those who sought care outside the home within 24 h of fever onset			
Public health facility	96	27.1	(22.8–31.9)
Village malaria worker	190	49.0	(44.1–54.0)
Private health facility	73	16.4	(13.2–20.3)
Pharmacy	14	2.5	(1.4–41.5)
Chemist/drug store	7	2.1	(0.9–4.2)
Grocery store	13	2.9	(1.6–4.9)
Reasons for choosing source of care^a^			
Proximity	499	77.5	(74.2–80.5)
Cost	202	29.3	(25.9–32.8)
Previous experience	236	39.6	(35.9–43.5)
Recommended provider	18	3.9	(2.4–6.1)
Quality of service	327	48.9	(45.1–52.7)
Friendliness of services	228	34.8	(31.2–38.5)
Availability of services	189	30.1	(26.6–33.8)
Trust in health provider	208	32.3	(28.8–36.0)

% of any variable based on < 10% missing

CI confidence interval

^a^Multiple response question

According to the qualitative findings, the basis for self-diagnosis was past experience with febrile illness and knowledge of malaria symptoms. One participant described:

*“When we get a fever, for the first day we are not sure yet what type of disease it could be. If the fever gets better by just taking paracetamol, then surely it is not malaria. But if the fever continues after having taken paracetamol, then it is clear that it is malaria.” [Forest-goer, 27 years, Ou Heng Village]*

Among the 28 forest-goers who participated in the IDIs, 15 (54%) sought care within 48 h and only three confirmed seeking care within 24 h. Seven respondents sought care after the 48 h mark and three did not seek care at all. According to one participant,

*“I waited 3 to 4 days before seeking any treatment. When I got chills that lasted for half an hour, I asked my wife to buy me some medicine. After I took the medicine, I got a bit better by sweating it out.” [Forest-goer, 28 years, Santrae Village]*

Results from the qualitative interviews also revealed that obtaining immediate care from a formal health facility was not possible on the weekends:

*“When I got a fever the last time, I could not get care from healthcare staff right away because it was on a Sunday, and health providers said they don’t work during the weekend and asked me to come back on Monday.”*

*[Forest-goer, age 25 years, Krasang Douslueng Village]*

### Sources of care

Village malaria workers were the most popular source of care, followed by public health facilities. Forty-nine percent of forest-goers who sought health services outside their home during their last febrile illness visited a VMW, 27% a public health facility, and 24% a private sector provider (Table 4). Forest-goers decided where to seek care based on several factors, including proximity (78%), perceived quality and effectiveness of care (49%), and previous experience with provider (40%). Other important factors that influenced decision-making about where to seek care included friendliness (35%) availability of services (30%), cost (30%), and trust (32%) (Table 6).

Ninety-four percent of forest-goers agreed that VMWs were available within their communities. At the same time, 92% also stated that a public health facility was available within their community. Fifty-nine percent of forest-goers confirmed the availability of a private health facility within their communities. According to one participant:

*“When I come back from the forest, I seek treatment for my febrile illness from the Village Malaria Worker.” [Forest-goer, 48 years, Voat Village]*

Among forest-goers who reported seeking care for their febrile illness within 24 h of onset, 24% reported seeking a second source of care. The decision to seek a second source of care was most often their own decision rather than by provider referral. Only 7% of forest-goers who sought care from a second source reported that they were referred by the previous source of care while the other 93% made the decision themselves.

### Behavioural determinants associated with care-seeking

#### Social norms

In bivariate analysis, the only significant behavioural determinant correlated with care-seeking within 24 h of developing a febrile illness was perceived community social norms. Among forest-goers who perceived that people within their community sought care within 24 h of developing a fever, 98% sought care within 24 h of developing a fever versus 88% of those who did not seek care immediately (Chisq = 7.05, p < 0.01) (Table 7).

**Table 7:** Bivariate analysis of behavioural determinants associated with care seeking outside the home within 24 h of febrile onset among forest-goers in Cambodia

	Sought care outside the home within 24 h of fever onset			Did not seek care outside the home within 24 h of fever onset			Chi-square statistic	p-value
	Unweighted sample (n)	Weighted population proportion	Bootstrapped 95% CI	Unweighted sample (n)	Weighted population proportion	Bootstrapped 95% CI		
Social norms—*perceived others in the community seek care within 24 h of developing a fever*								
Yes	135	97.6	(90.9–99.4)	460	87.7	(83.4–90.9)	7.05	0.01
No	3	2.3	(0.06–9.1)	56	12.3	(9.0–16.6)		

CI confidence interval

Findings from the weighted logistic regression models (Table 8) showed that after controlling for confounders and covariates, the odds of seeking care within 24 h of developing a fever was 5 times greater for forest-goers who perceived that care-seeking for a fever within 24 h of onset was a normative behaviour in the community compared to those who did not perceive care-seeking as a normative behaviour in the community (OR: 4.9, 95% CI 1.32–18.12, p-value: 0.02).

**Table 8:** Weighted crude and adjusted odds ratio and 95% confidence intervals of care-seeking withing 24-h of developing a fever on perceived community social norms

	Unadjusted OR	95% CI	P-value	Adjusted OR^a^	95% CI	p-value
Perceived community social norms						
Yes	5.36	(1.64–17.4)	0.01	4.91	(1.32–18.12)	0.02
No (ref)						

OR odds ratio, CI confidence interval

^a^Controlled for age, sex, marital status, education, ID poor, exposure to malaria social behaviour change campaign within the past 6 months, source of care (e.g., public vs. private), and perceived quality of care

## Discussion

This study presents findings from a mixed-methods study of malaria KAP among forest-goers in Cambodia and behavioural determinants correlated with two important malaria behaviours, LLIN/LLIHN use and care-seeking within 24 h of fever onset. Findings indicated that study participants had some knowledge about malaria and recommended prevention practices. Most forest-goers owned an LLIN/LLIHN and were able to bring it to the forest. While nearly all study participants perceived LLIN/LLIHN use as an important preventative measure to decrease risk of malaria, only three quarters of study participants reported regularly sleeping under an LLIN/LLIHN during their last visit to the forest. This finding is similar to findings from other studies in the region [22–24]. For example, a study along the Thai-Myanmar border found that although 74% of households owned an ITN, only 53% used it every day [25]. Consistent with other studies on mosquito net use, the most widely identified reason forest-goers did not use an LLIN/LLIHN during their last visit to the forest was discomfort due to heat [26, 27]. Heat was also frequently mentioned as a deterrent of LLIN/LLIHN use in the qualitative data. These findings underscore the importance of alternative vector control tools that are appropriate for use by forest-goers during forest visits.

Study findings on care-seeking behaviours were concerning. Only 21% of all study participants were early care-seekers, seeking care in 24 h or less. Twenty percent were delayed care-seekers, seeking care between 24 and 48 h, 16% were wait and see care-seekers, seeking care more than 48 h after developing a fever, and 43% did not seek care at all. In total, 57% of all study participants sought care outside their home during their last febrile illness event. This figure is substantially below national and international targets aimed at identifying and testing all fevers for malaria. The main reason participants did not seek care was low perceived risk. Furthermore, among those who did seek care, only 39% did so within the recommended 24 h. Findings from the qualitative interviews clarified that forest-goers often evaluated the severity of their illness based on past experiences and preferred to self-treat and see if the fever resolved before seeking formal care. These findings are consistent with other research which shows that forest-goers undergo a complex decision-making process, weighing the perceived severity of their illness against time spent accessing care and the opportunity cost involved [28, 29].

Nearly all study participants reported that health services were available in their community with 49% preferring to seek care from a VMW or community provider. This is in contrast to other studies in the Greater Mekong Sub-region that found that forest-goers preferred services in the private sector due to service quality and accessibility [25, 30]. In Cambodia, the combined effect of a strengthened VMW programme and proximity to community-based services is likely a strong factor in the preferences observed in this study. Trust and proximity were cited as significant factors in provider choice.

The key findings from the cross-sectional survey revealed that forest-goers who perceived LLIN/LLIHN use as a normative behaviour in the community were 2.7 times more likely to use an LLIN/LLIHN during their last visit to the forest compared to those who did not perceive LLIN/LLIHN use as a normative community behaviour (OR: 2.69, 95% CI 1.99–3.64), and forest-goers who perceived that care-seeking within 24 h of fever onset was a normative behaviour in the community were five times more likely to seek care themselves within 24 h of fever onset compared to forest-goers who did not perceive timely care-seeking for a fever as a normative community behaviour (OR: 4.91, 95% CI 1.32–18.12).

These findings corroborate findings from studies on other health topics that show a positive relationship between perceived normative behaviour and individual behaviour [31, 32]. For malaria-related behaviours, several studies investigating ideational predictors of net use and care-seeking also reported positive correlations with social norms. In Nigeria and Madagascar, for example, female care-givers for children under-5 who perceived that it was the community norm to promptly seek care for children with fever were significantly more likely to seek care than those who did not perceive care-seeking for fever as a normative community behaviour [33]. Likewise, in Madagascar, the belief that bed net use was a community norm was a positive predictor of net use [23].

This study’s findings underscore the importance of social influence on individual behaviour. As such, identifying and highlighting social norms should be a central focus of future SBC activities for malaria elimination in Cambodia. For example, a community-wide SBC activity targeting forest-goers and their social networks could be implemented, disseminating messages that highlight social norms related to malaria prevention and care-seeking as normative community behaviours [34]. Other methods such as the positive deviance approach or popular opinion leader model have been effective at increasing LLIN/LLIHN use in remote communities, especially in communities that do not have access to TV or radio [22, 35]. Using such approaches could be effective in this context as well [36].

Reinforcing knowledge and practices around prompt care seeking for all fevers, irrespective of perceived severity is crucial. Social behaviour change activities should be paired with interventions that address structural barriers to care-seeking. For example, it is important that forest-goers retain access to malaria interventions while in the forest, especially because malaria elimination is predicated on early diagnosis and treatment. Given that VMWs were the preferred source of care and a trusted source of information, interventions that invest in further strengthening service communication skills of VMWs and ensuring they are equipped with sufficient/enough tools to treat and counsel patients could also be beneficial [37]. Finally, programmes should promote moving from a solely individual response to a collective community response aimed at achieving malaria elimination. Recent research has shown that collective community response can be an effective tool for malaria elimination when communities are part of decision-making processes, feel equipped and empowered to take control over their own health, and have built trusting relationships with programme staff [38]. Studies have also shown that community-based proactive case detection reduces symptomatic malaria prevalence [39, 40].

This study is not without limitations. A main limitation of this study was the cross-sectional nature of the data which limits the ability to draw conclusions about causality. Another limitation is that the data were based on self-report and subject to social desirability bias which could lead to over or under reporting of specific behaviours such as LLIN/LLIHN use and care-seeking. Compensation is an important component of RDS, but it can be challenging to ensure an appropriate amount is provided. To ensure that compensation was not coercive the research team set amounts according to the going rate in the country. The correlation between care-seeking and perceived social norms should be interpreted with caution given the wide 95% confidence interval. Findings from this study are not generalizable to the larger forest-goer population given that the study was among those who had a febrile illness within 3 months prior to the study. Strengths of the study included the use of RDS methods which allowed access to forest-goers who are a hard-to-reach population due to their mobility and migratory patterns. Additionally, the study used a mixed-methods approach, supplementing the quantitative data with qualitative IDIs which provided for a more complete understanding of forest-goers’ perceptions and opinions on malaria prevention and care-seeking behaviours. Finally, the study adds to the limited body of literature on forest-goers’ malaria risk behaviours and determinants of those behaviours.

## Conclusion

Individuals that work and sleep in the dense and remote forests of Cambodia remain at high risk of acquiring and transmitting malaria. This study’s findings suggested that social norms and social support were important behavioural determinants of LLIN/LLIHN use while in the forest and care-seeking within 24 h of developing a febrile illness. As Cambodia continues toward the goal of malaria elimination, SBC activities targeting forest-goers should encourage and promote positive social norms, community norms, and social support to increase consistent use of LLIN/LLIHNs and promote timely care-seeking on fever onset.

## Data Availability

The datasets used and/or analysed during the current study are available from the corresponding author on reasonable request.

## Abbreviations

CNM: National Center for Parasitology, Entomology and Malaria Control
IDI: In-depth interview
ITN: Insecticide-treated net
GMS: Greater Mekong Sub-region
KAP: Knowledge, attitudes and practices
LLIN: Long-lasting insecticidal net
LLIN: Long-lasting insecticidal hammock net
MEAF: Malaria Elimination Action Network
MMP: Mobile migrant populations
PMI: Presidents Malaria Initiative
PSI: Population Services International
RDS: Respondent driven sampling
SBC: Social behaviour change
VMW: Village malaria worker

## References

[1] Sovannartoh S. Achievements and plan for 2021 of the National Malaria Control Program. In: The 38th annual congress of the National Center for Parasitology, Entomology and Malaria Control, Cambodia; 2021.

[2] Durnez L, Mao S, Denis L, Roelants P, Spchantha T, Coosemans M (2013). Outdoor malaria transmission in forested villages of Cambodia. Malar J.

[3] National Center for Parasitology, Entomology and Malaria Control. Malaria elimination action framework (MEAF) 2016–2020. Cambodia: Cambodia Ministry of Health; 2015.

[4] Mekong malaria elimination program epidemiology summary. 2022.

[5] Kunkel A, Nguon C, Iv S, Chhim S, Peov D, Kong P (2021). Choosing interventions to eliminate forest malaria: preliminary results of two operational research studies inside Cambodia forests. Malar J.

[6] Guyant P, Canavati SE, Chea N, Ly P, Whittaker M, Roca-Feltrer A (2015). Malaria and the mobile migrant population in Cambodia: a population movement framework to inform strategies for malaria control and elimination. Malar J.

[7] Koenker H, Kilian A, Hunter G, Acosta A, Scandurra L, Fagbemi B (2015). Impact of a behaviour change intervention on long-lasting insecticidal net care and repair behaviour and net condition in Nasarawa State, Nigeria. Malar J.

[8] The Health Communication Capacity Collaborative. Malaria SBCC evidence and literature review. Baltimore: Johns Hopkins Center for Communication Programs; 2017.

[9] Canavati SE, Zegers de Beyl C, Ly P, Shafique M, Boukheng T, Rang C (2016). Evaluation of intensified behavior change communication strategies in an artemisinin resistance setting. Malar J.

[10] Boulay M, Lynch M, Koenker H (2014). Comparing two approaches for estimating the causal effect of behaviour-change communication messages promoting insecticide-treated bed nets: an analysis of the 2010 Zambia malaria indicator survey. Malar J.

[11] Chung A, Case P, Gosling J, Gosling R, Madinga M, Chikodzore R (2020). Scaling up malaria elimination management and leadership: a pilot in three provinces in Zimbabwe. Malar J.

[12] The RBM Partnership to End Malaria. The strategic framework for malaria social and behaviour change communication 2018–2030.

[13] Vastine A, Gittelsohn J, Ethelbah B, Anliker J, Caballero B (2005). Formative research and stakeholder participation in intervention development. Am J Health Behav.

[14] Heckathron D (1997). Respondent driven sampling: a new approach to the study of hidden populations. Soc Probl.

[15] Johnston L, Hakim A, Dittrich S, Burnett J, Kim E, White R (2016). A systematic review of published respondent-driven sampling surveys collecting behavioral and biologic data. AIDS Behav.

[16] Gile K, Johnston L, Salganik M (2015). Diagnostics for respondent-driven sampling. J R Stat Soc.

[17] Paudel M, Tesfazghi K, Nguyen H, Phok S, Srinivasan A, Wheeler J (2021). The use of respondent-driven sampling to assess febrile illness treatment-seeking behaviours among forest-goers in Cambodia and Vietnam. Malar J.

[18] IDPoor Program 2022. https://www.idpoor.gov.kh/about/process.

[19] Hirschhorn L, Krasne M, Maisonneuve J, Kara N, Kalita T, Henrich N (2018). Integration of the opportunity-ability-motivation behavior change framework into a coaching0based WHO Safe Childbirth Checklist program in India. Int J Gynaecol Obstet.

[20] Hosmer DW, Lemeshow S, Sturdivant RX (2013). Applied logistic regression.

[21] Hsieh HF, Shannon S (2005). Three approaches to qualitative content analysis. Qual Health Res.

[22] Acosta A, Koenker H, Brown A, Bertram K, Blaufuss S, Filemyr E (2019). Social and behavior change for insecticide-treated nets.

[23] Storey D, Babalola S, Ricotta E, Fox K, Toso M, Lewicky N (2018). Associations between ideation variables and bed net use in Madagascar, Mali, and Nigeria. BMC Public Health.

[24] WHO (2017). Achieving and maintaining universal coverage with long-lasting insecticidal nets for malaria control.

[25] Nofal S, Peto T, Adhikari B, Tripura R, Callery J, Bui TM (2019). How can interventions that target forest-goers be tailored to accelerate malaria elimination in the Greater Mekong Subregion? A systematic review of the qualitative literature. Malar J.

[26] Pulford J, Hetzel M, Bryant M, Siba P, Mueller I (2011). Reported reasons for not using a mosquito net when one is available: a review of the published literature. Malar J.

[27] Gunasekaran K, Sahu SS, Vijayakumar KN, Jambulingam P (2009). Acceptability, willing to purchase and use long lasting insecticide treated mosquito nets in Orissa State, India. Acta Trop.

[28] Jongdeepaisal M, Ean M, Heng C, Buntau T, Tripura R, Callery J (2021). Acceptability and feasibility of malaria prophylaxis for forest goers: findings from a qualitative study in Cambodia. Malar J.

[29] Pooseesod K, Parker D, Meemon N, Lawpoolsri S, Singhasivanon P, Sattabongkot J (2021). Ownership and utilization of bed nets and reasons for use or non-use of bed nets among community members at risk of malaria along the Thai-Myanmar border. Malar J.

[30] Shafique M, Edwards HM, De Beyl CZ, Thavrin BK, Min M, Roca-Feltrer A (2016). Positive deviance as a novel tool in malaria control and elimination: methodology, qualitative assessment, and future potential. Malar J.

[31] Chung A, Rimal R (2016). Social norms: a review. Rev Commun Res.

[32] Latkin CA, Kuramoto SJ, Davey-Rothwell MA, Tobin KE (2010). Social norms, social networks, and HIV risk behavior among injection drug users. AIDS Behav.

[33] Do M, Babalola S, Awangtang G, Toso M, Lewicky N, Tompsett A (2018). Association between malaria-related ideational factors and care-giving behavior for fever among children under five in Mali, Nigeria, and Madagascar. PLoS ONE.

[34] Perkins J, Krezanoski P, Takada S, Kakuhikire B, Batwala V, Tsai A (2019). Social norms, misperceptions, and mosquito net use: a population-based, cross-sectional study in rural Uganda. Malar J.

[35] Koenker H, Keating J, Alilio M, Acosta A, Lynch M, Nafo-Traore F (2014). Strategic roles for behavior change communication in a changing malaria landscape. Malar J.

[36] Shafique M, George S. Positive deviance: an asset-based approach to improve malaria outcomes. Malaria Consortium; 2014.

[37] Canavati SE, Lawpolsri S, Quintero C, Nguon C, Ly P (2016). Village malaria worker performance key to the elimination of artemisinin-resistant malaria: a Western Cambodia health system assessment. Malar J.

[38] Watanabe N, Kaneko A, Yamar S, Taleo G, Tanihata T (2015). A prescription for sustaining community engagement in malaria elimination on Anetiyum Island, Vanatu: an application of Health empowerment. Malar J.

[39] Lek D, Callery JJ, Nguon C, Debackere M, Sovannaroth S, Tripura R (2020). Tools to accelerate falciparum malaria elimination in Cambodia: a meeting report. Malar J.

[40] Kheang ST, Sovannaroth S, Barat LM, Dysoley L, Kapella BK, Po L (2020). Malaria elimination using the 1-3-7 approach: lessons from Sampov Loun, Cambodia. BMC Public Health.

